# Targeting Epigenetic Modifiers Can Reduce the Clonogenic Capacities of Sézary Cells

**DOI:** 10.3389/fonc.2021.775253

**Published:** 2021-10-26

**Authors:** Alain Chebly, Martina Prochazkova-Carlotti, Yamina Idrissi, Laurence Bresson-Bepoldin, Sandrine Poglio, Chantal Farra, Marie Beylot-Barry, Jean-Philippe Merlio, Roland Tomb, Edith Chevret

**Affiliations:** ^1^ Univ. Bordeaux, INSERM U1053, Bordeaux Research in Translational Oncology (BaRITOn), Bordeaux, France; ^2^ Medical Genetics Unit (UGM), Faculty of Medicine, Saint Joseph University, Beirut, Lebanon; ^3^ Department of Genetics, Hotel-Dieu de France Medical Center, Beirut, Lebanon; ^4^ Department of Dermatology, Bordeaux University Hospital Center, Bordeaux, France; ^5^ Tumor Bank and Tumor Biology Laboratory, Bordeaux University Hospital Center, Pessac, France; ^6^ Department of Dermatology, Hotel-Dieu de France Medical Center, Beirut, Lebanon

**Keywords:** cutaneous T-cell lymphomas (CTCL), Sezary syndrome (SS), 5-azacytidine, romidepsin, vorinostat, Histone deacetylase inhibitor, DNA methyltransferase inhibitor, TERT

## Abstract

Sézary syndrome (SS) is an aggressive leukemic variant of cutaneous T-cell lymphomas (CTCL) in which the human Telomerase Reverse Transcriptase (*hTERT*) gene is re-expressed. Current available treatments do not provide long-term response. We previously reported that Histone deacetylase inhibitors (HDACi, romidespin and vorinostat) and a DNA methyltransferase inhibitor (DNMTi, 5-azacytidine) can reduce *hTERT* expression without altering the methylation level of *hTERT* promoter. Romidepsin and vorinostat are approved for CTCL treatment, while 5-azacytidine is approved for the treatment of several hematological disorders, but not for CTCL. Here, using the soft agar assay, we analyzed the functional effect of the aforementioned epidrugs on the clonogenic capacities of Sézary cells. Our data revealed that, besides *hTERT* downregulation, epidrugs’ pressure reduced the proliferative and the tumor formation capacities in Sézary cells *in vitro*.

## Introduction

Sézary syndrome (SS) is a rare leukemic variant of cutaneous T-cell lymphoma (CTCL) characterized by a malignant monoclonal proliferation of mature CD4+ T-cells named Sézary cells (1) for which current available treatments do not provide long-term responses (2). The etiology of the molecular oncogenesis of SS are still not fully understood, therefore, molecular targets for directed therapeutic interventions remain to be defined (3). Hence, there is a need for additional effective therapeutic options.

Data available from genomic studies provide rational evidences for implementing epigenetic therapies in SS patients, through DNA demethylating agents and histone deacetylase inhibitors (HDACi) (3). Histone deacetylase (HDAC) are a class of widely expressed enzymes that not only can detach the acetyl group from histones, but can also deacetylate a large variety of non-histone proteins whose activities rely on their acetylation status (4–6). The non-histone proteins regulated by HDAC are involved in various pathways that may be implicated in lymphoproliferative disorders (5, 6). Indeed, reduced acetylation is associated with enhanced proliferation and survival of lymphoma cells, while increased acetylation is associated with tumor growth arrest and cell death (7). To date, human HDAC comprise a family of 18 enzymes, grouped in four classes (4, 7, 8). HDACi can target several classes of HDAC enzymes (pan-inhibitors) or selectively inhibit one class of HDAC enzymes. HDACi have been shown to be of benefit for patients with hematological malignancies. Two HDACi, romidepsin (specific inhibitor against HDAC class I) and vorinostat (inhibitors), were approved by the Food Drug Administration (FDA) for the treatment of relapsed/refractory CTCL in 2009 and 2006, respectively (9, 10). HDACi are known for their cytotoxicity which distinguishes tumor cells from normal cells (11), although it remains unclear why tumor cells are more sensitive to HDACi-induced cell death than normal cells. HDACi may influence cell cycle progression, apoptosis and angiogenesis, however, the exact mechanism of action of HDACi in T-cell lymphomas remains unknown to date (12). Besides HDACi, other drugs belonging to the DNA packaging modifiers such as DNA methyltransferase (DNMT) inhibitors (DNMTi), 5-azacytidine and 5-aza-2’-deoxycytidine (Decitabine, DAC) are available. These DNMTi are FDA-approved for the treatment of myelodysplastic syndrome (MDS) and acute myeloid leukemia (AML), but not for the treatment of CTCL.

DNA packaging modifiers have been thoroughly investigated, as reported in the literature, at both cellular and whole genomic level. Our team, on the other hand, examined the effect of these DNA packaging modifiers at the level of a specific gene promoter sequence, the human telomerase reverse transcriptase gene (*hTERT*). We had previously demonstrated that in SS, the telomerase (*hTERT*) expression holds a critical role in telomeres maintenance and tumorigenesis (13, 14). We then reported that each of romidepsin, vorinostat and 5-azacytidine can reduce *hTERT* expression while maintaining *hTERT’*s promotor methylation status (14). We herein, report on the effect of the aforementioned DNA packaging modifiers on the tumorigenic capacities of Sézary cells *in vitro*, along with the changes in *hTERT* gene expression.

## Methods

In this study, we focused on SS an aggressive leukemic form of CTCL. Three SS cell lines were investigated: one commercially-available cell line HuT78 (ATCC, France) and two cell lines (SS patient-derived-cells): L2 and L4 (15) recently developed and well-characterized at our laboratory. Cells were cultured as previously reported (14, 15). The half maximal inhibitory concentrations (IC50) for 5-azacytidine or HDACi were determined at 72h or 48h, respectively, using the luminescent cell viability assay CellTiter-Glo^®^ (Promega, USA). SS cells colony-formation capacity (clonogenicity) was determined using soft agar assay (50.000 cells per well), after releasing the DNMTi or HDACi pressure. Statistical analyses were performed using the GraphPad Prism software (version 8.4). All analyses were performed in biological and technical triplicates.

## Results and Discussion

First, we investigated the effect of the hypomethylating agent 5-azacytidine on *hTERT* promoter methylation status and *hTERT* expression. In accordance with our previous findings, all Sézary cells analyzed expressed the telomerase. In order to treat these cells with epidrugs, we first determined their IC50. The IC50 values of 5-azacytidine after 72h in HuT78, L2 and L4 Sézary cells were 3nM, 1.7nM and 2.3nM, respectively. Consequently, Sézary cells were treated with the aforementioned IC50 conditions. In all treated cells (TC), a decrease in *hTERT* mRNA levels occurred with no change in the methylation status of *hTERT* promoter. This result is in compliance with our initial observations (14) and in line with previous findings in the literature where DNMTi (5-azacytidine or decitabine) have been reported to alter the expression of some genes (other than *hTERT*) without changing their promoter methylation status (16). This effect may result from demethylation of upstream genes (like transcription factors) or regulatory elements (like enhancers) or from secondary responses to DNA damage or repair mechanism caused by DNMTi toxicity (16).

**Figure 1:**
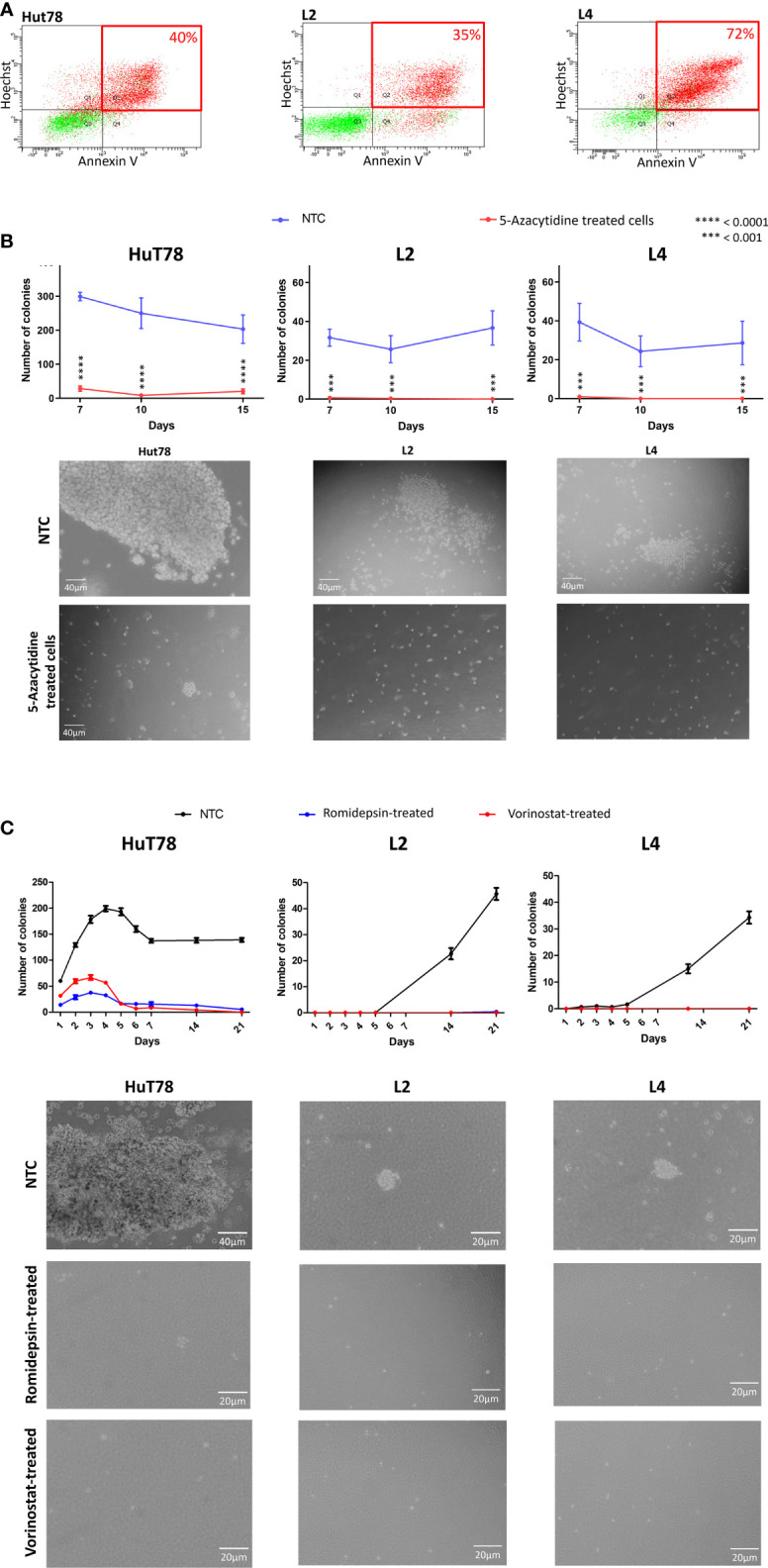
5-azacytidine, romidepsin and vorinostat treatment in Sézary cells *in vitro*. **(A)** Flow cytometry plots showing the apoptosis/necrosis analyses of 5-azacytidine treated cells and displaying the % of late apoptotic cells for HuT78, L2 and L4 (40%, 35% and 72% respectively). **(B)** Graphs showing the number of colonies per well in NTC (blue) and 5-azacytidine TC (red); and microscopic captures of the colonies observed in NTC (upper images) and after 5-azacytidine treatment (lower images), of HuT78, L2 and L4 Sézary cell lines. **(C)** Colonies formation in HuT78, L2 and L4 without treatment (NTC, black line) or after 48h of romidepsin (blue line) or vorinostat (red line); and microscopic captures of the colonies observed in NTC (first line) and after treatment (second and third lines), for HuT78, L2 and L4 Sézary cell lines.

In our study, apoptosis/necrosis analyses by flow cytometry using AnnexinV/Hoescht assay revealed that a majority of Sézary TC with 5-azacytidine were in late apoptosis (**Figure 1A**), suggesting that TC were engaged in a death program possibly related to 5-azacytidine treatment (17). Similarly, Rozati et al. had previously reported that 5-azacytidine in combination with romidepsin synergistically induced apoptosis in CTCL cells *in vitro* (18).

We further investigated the effect of 72h 5-azacytidine treatment on Sézary cells colony-forming potential using the soft agar assay. This assay enables the evaluation of the transformed and carcinogenic cells’ ability to grow and divide independently of their surrounding environment (19). In our experimental protocol, drugs’ pressure was released in the soft agar assay. While in HuT78 non treated-cells (NTC), the number of colonies per well was 299 at day 7 and 203 at day 15, this number was significantly lower in 5-azacytidine TC (*P*<0.0001) with 28 colonies per well at day 7 and 20 at day 15 (**Figure 1B**). Looking through the microscope revealed that Hut78 NTC colonies were countless and huge, while they looked smaller with unhealthy appearance in Hut78 TC (**Figure 1B**). Similarly, in L2 NTC, the number of colonies per well was 32 at day 7 and 37 at day 15. This number significantly decreased (*P*<0.001) in 5-azacytidine TC to 1 per well at day 7 and 0 at day 15 (**Figure 1B**). Also, in L4 NTC, the number of colonies per well was 39 at day 7 and 29 at day 15 then it dropped down (*P*<0.001) in L4 TC to 1 per well at day 7 and 0 at day 15 (**Figure 1B**). Overall, our observations showed that 72 hours of 5-azacytidine treatments, induced an inhibition of colony formation in L2 and L4 cells and drastically reduced the capacity to form colonies in HuT78 cells (**Figure 1B**). It has been reported that DNMTi have the potential to reduce the clonogenicity of various cancer cells, including leukemia, colorectal cancer, uveal and skin melanoma (20–22). Our data demonstrate a similar effect in Sézary cells. Furthermore, Sézary TC showed limited proliferative capacity during the 15 days after treatment suggesting that following the massive cell death induced by 5-azacydine, the surviving cells experienced a reduced proliferative capacity. We are aware that the molecular pathway implicated in this response to treatment remains to be elucidated, however these data represent a starting point for further functional analyses.

To complete our investigation on epidrugs, we compared DNMTi and HDACi effects on Sézary cells’ clonogenic capacities (**Table 1**). With this aim in mind, we treated SS cells with romidepsin or vorinostat using IC50 values (Romidepsin: 1.56nM, 1.85nM and 21.40nM; Vorinostat: 0.254µM, 0.830µM and 2.44µM; in HuT78, L2 and L4, respectively) and we evaluated first the effect of HDACi on *hTERT* expression. Consistent with our previous finding, *hTERT* promoter methylation status remained unaffected by HDACi pressure (14) even though we observed a drop in *hTERT* expression. Indeed, compared to NTC, the *hTERT* expression levels in HuT78 decreased by 37% with romidepsin and 36% with vorinistat. In L2, *hTERT* expression levels declined by 56% with romidepsin and 83% with vorinostat. In L4, *hTERT* expression levels were reduced by 84% with romidepsin and 91% with vorinostat.

**Table 1:** Summary of epidrugs’ effects in Sézary cells *in vitro*.

Drug	Family	Impact on Sézary cells *in vitro*			
		*hTERT* expression	*hTERT*promoter methylation	Proliferation	Clonogenicity
**5-azacytidine**	DNMTi	Decreased	Unchanged	Decreased	Decreased
**Romidepsin**	Class I HDACi	Decreased	Unchanged	Decreased	Decreased
**Vorinostat**	Class I, II and IV HDACi	Decreased	Unchanged	Decreased	Decreased

*DNMTi, DNA MethylTransferase inhibitor; HDACi, Histone DeACetylase inhibitor; hTERT, human TElomerase Reverse Transcriptase.

In order to evaluate the impact of HDACi on the *in vitro* tumorigenic cell capacities, Sézary cells were treated for 48h either with romidepsin or vorinostat prior to be cultured in soft-agar wells without any drug pressure. In these experiments, similar impacts on the clonogenic capacities were observed in all Sézary cell lines. Indeed, Sézary cells’ clonogenic capacities were drastically reduced (**Figure 1C**). Compared to NTC, HuT78 colonies observed in romidepsin and vorinostat TC were smaller in size and less numerous with almost zero colonies at day 21 (**Figure 1**). L2 and L4 TC either with romidepsin or vorinostat were unable to form colonies even at day 21 (0 to 1 colony per well were counted) (**Figure 1C**). Altogether, we have obtained comprehensive results proving that HDACi agents have the potential to reduce clonogenic and proliferation capacities of Sézary cells (**Table 1**). Our observations are consistent with previous findings where HDACi agents were reported to impact the clonogenicity of cancer cells, such as romidepsin in bladder cancer (23) and vorinostat in polycythemia vera (PV) hematopoietic progenitors expressing JAK2^V617F^ (24). The *hTERT* gene expression was also reported to impact the clonogenicity of CTCL tumor cells (13). Additionally, our results suggest, for the first time, a role for HDACi in reducing the clonogenic capacities of Sézary cells by decreasing *hTERT* expression. It is interesting to note that the slow-down in proliferation observed in HDACi TC can be linked to the cell cycle arrest and to the induction of apoptosis-related genes in response to the HDACi treatments. Although our data do not provide mechanistic investigations of the molecular basis that could explain HDACi effect on the clonogenic and proliferative capacities of Sézary cells, however, it is well documented in the literature that the growth arrest and/or apoptosis induced by HDACi in G1 phase could depend on cyclin-dependent kinase (CDK) inhibition (25). It is also reported that HDACi may block the S phase progression by repressing two genes involved in DNA synthesis, CTP synthase and thymidylate synthase (26, 27), or induce a G2/M phase arrest by activating a G2-phase checkpoint in the presence of aberrantly acetylated centromeres (28). Furthermore, HDACi were reported to induce global acetylation and DNA damages leading to cell death in apoptosis-susceptible CTCL cells (27, 29), which correlates very well with the massive cell death observed in our study in HDACi treated Sézary cells, and with the cell-death persistence despite the drug’s pressure release.

## Conclusion

Recent experiments conducted in the commercially available Sézary cell line, HuT78, showed that one epigenetic agent (either DNMTi or HDACi) may reduce cellular viability and exert growth inhibition mostly by inducing apoptosis (18, 30). Our results, using also HuT78 and two other newly-developed Sézary cell lines derived from SS patients (15), reinforce the findings reported above regarding DNMTi or HDACi treatments. Moreover, our results offer unprecedented evidence for functional consequences of epidrugs treatments on tumor formation capacities in Sézary cells *in vitro.* Our previous work showed that modulating *hTERT* by lentivirus can modify the clonogenic capacities of CTCL cells (13). Indeed, the most remarkable observation stemming from the present investigation is that the changes in Sézary cells’ clonogenic capacity induced by epidrugs correlate strongly with *hTERT* expression. This situation has been previously evoked by Qu et al.’s who had stated that DNMTi and HDACi may alter gene expression by dynamic modifications of chromatin accessibility at key regions of tumorigenic gene promoters (31), and this may be similar to *hTERT* gene and its regulators (activators or repressors).

It has been reported that *hTERT* promoter does not behave in a simplistic way. Both, our previous study and the current one, reinforce this hypothesis and represent a step forward towards a better understanding of the relationship between targeting epigenetic modifiers in cancer and the response generated at the molecular level such as *hTERT* gene and also at the cellular level.

## Data Availability Statement

The original contributions presented in the study are included in the article/supplementary material. Further inquiries can be directed to the corresponding author.

## Ethics Statement

This study was approved by the institutional review board at Bordeaux University, in accordance with the Declaration of Helsinki on ethical principles for medical research involving human subjects.

## Author Contributions

Conceptualization: AC and EC. Methodology: AC, MP-C, LB-B, YI, and EC. Formal Analysis and investigations: AC, MP-C, YI, and EC. Resources: AC, MP-C, and EC. Data Curation: AC, MP-C, CF, and EC. Writing – Original Draft Preparation: AC. Visualization: AC. Writing – Review & Editing: EC, SP, LB-B, CF, J-PM, MB-B, and RT. Funding Acquisition: EC. Supervision: J-PM and EC. All authors contributed to the article and approved the submitted version.

## Funding

This work was supported by grants from the National Institute of Health and Medical Research (INSERM), the French Society of Dermatology (SFD), La Ligue Contre le Cancer Gironde and the research council at Saint Joseph University of Beirut (Grant no. FM412). AC was supported by grants from Hubert Curien Partnership (PHC-CEDRE) and ERASMUS+.

## Conflict of Interest

The authors declare that the research was conducted in the absence of any commercial or financial relationships that could be construed as a potential conflict of interest.

## Publisher’s Note

All claims expressed in this article are solely those of the authors and do not necessarily represent those of their affiliated organizations, or those of the publisher, the editors and the reviewers. Any product that may be evaluated in this article, or claim that may be made by its manufacturer, is not guaranteed or endorsed by the publisher.
